# Amidation Reaction System: Kinetic Studies and Improvement by Product Removal

**DOI:** 10.1021/acsomega.1c03843

**Published:** 2021-11-05

**Authors:** Issadaporn Wongwanichkangwarn, Sunun Limtrakul, Terdthai Vatanatham, Palghat A. Ramachandran

**Affiliations:** † Department of Chemical Engineering, Faculty of Engineering, Kasetsart University, Bangkok 10900, Thailand; ‡ Center of Excellence on Petrochemical and Materials Technology, Department of Chemical Engineering, Faculty of Engineering, Kasetsart University, Bangkok 10900, Thailand; § Center for Advanced Studies in Industrial Technology, Faculty of Engineering, Kasetsart University, Bangkok 10900, Thailand; ∥ Department of Energy, Environmental & Chemical Engineering, Washington University in St. Louis, St. Louis, Missouri 63130, United States

## Abstract

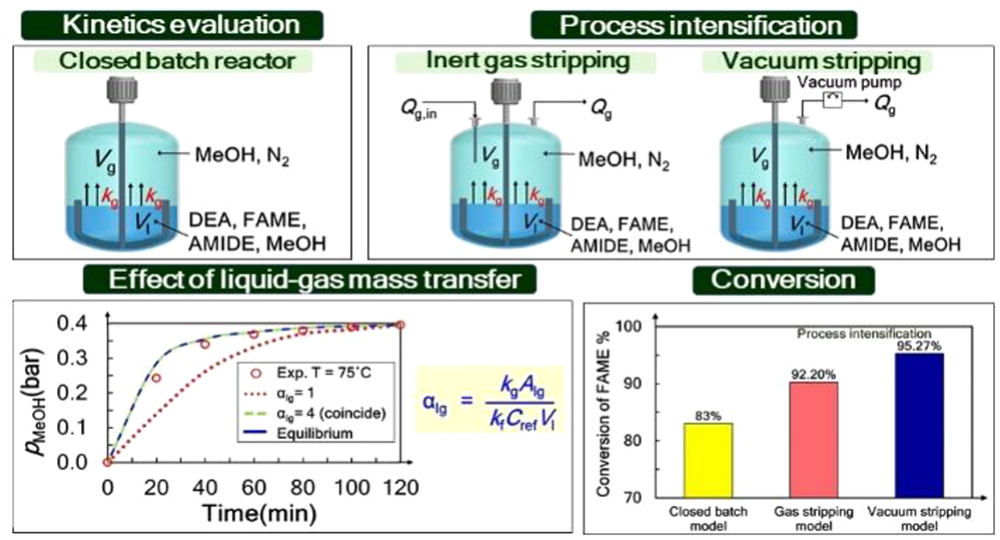

The amidation reaction to produce fatty acid diethanolamide is an important unit process to produce surfactants from renewable sources rather than from petroleum sources. Amidation is a liquid-phase reaction between diethanolamine with a fatty acid methyl ester. Since the reaction is reversible, the conversion is limited by equilibrium, the side product being methanol, which is volatile. Hence, mass transfer effects need to be considered in the interpretation of kinetic data. Further, the elimination of methanol can help to shift the reaction forward. Thus, the process has the potential for process intensification. This paper provides a batch reactor model to interpret the simulation data and includes mass transfer effects analyzed using a dimensionless mass transfer parameter (α_lg_). Using values of this parameter greater than 4 leads to an equilibrium model where the methanol partial pressure in the bulk gas approaches that at the interface. Using this model, the kinetic and equilibrium parameters for the amidation reaction were determined using experimental data in the first part of this study. The experimental data for fitting the parameters are obtained from a closed batch reactor operated with an initial pressure of 1 bar and a temperature range of 70–80 °C. The second part of the paper examines two process-intensification concepts—*viz*., inert gas and vacuum stripping of methanol from the reactor—and simulates the process in the form of mass-transfer-based models. Improvement in the final conversion was demonstrated in both approaches, and predictions of the vacuum stripping model are in good agreement with the experimental results. Thus, the developed vacuum stripping model is useful for accurate analysis and design of a reactor with vacuum stripping. The novelty of the work is obtaining rate and reaction equilibrium constants, enthalpy of reaction, and liquid activity coefficient for amidation, which have no prior reporting, and providing the viability of options for side product removal. The applied modeling approaches and the experimental facilities and methods are established.

## 1 Introduction

Surfactants, also known as surface-active compounds, are able to reduce the surface tension between two liquid phases that have dissimilar polarities such as water/oil or oil/water. These chemicals can thus be used as foaming, emulsifying, and adhesive agents and find various applications as follows: cosmetics, detergents, textiles, polymers, paints, agrochemicals, pharmaceuticals, and lubricants.^1−3^ Currently, these surfactants are usually synthesized from nonbiodegradable petroleum derivatives.^4^ Surfactants derived from biodegradable raw materials—*e*.*g*., coconut oil, palm oil, soybean oil, sunflower oil, and jatropha oil^5−10^—should be ecologically more acceptable. In particular, fatty acid diethanolamide is a versatile class of surface-active agents widely used in many industrial applications such as detergents, coatings, paints, dyes, and metal-working fluids. Fatty acid diethanolamide is a product of the liquid-phase reaction between diethanolamine with a fatty acid methyl ester. This reaction is called amidation and is represented in eq 1.
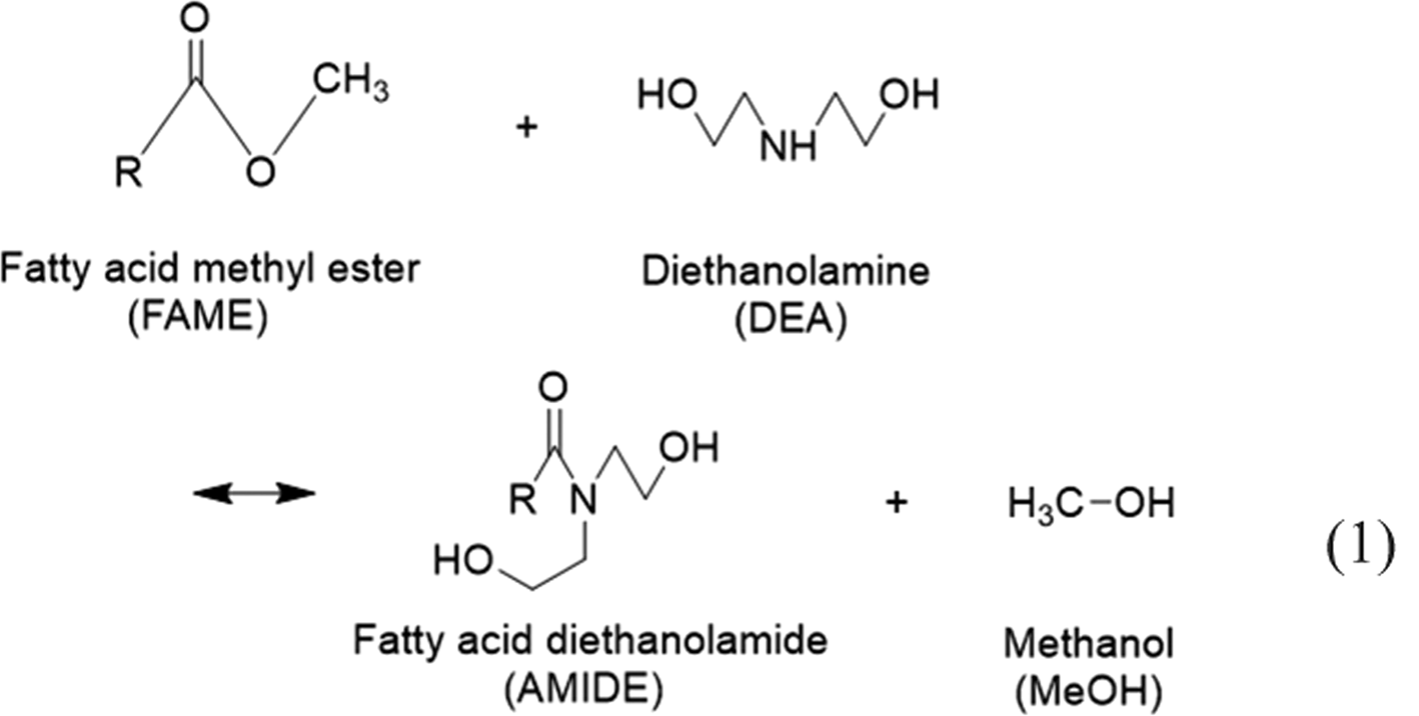
1

Certain parameters—namely, equilibrium and reaction rate constants—of amidation are significant for the analysis and design of amidation reactors. Only a few studies have reported the literature on the kinetics of this reaction.^11,12^ These authors studied the kinetics of amidation at high temperatures: at 100–160 °C. At a high temperature, the occurrence of byproducts such as an overcondensate and ester with a free amine can be found. Therefore, a medium-range of temperature is selected in this kinetic study, thus limiting byproduct formation.

The equilibrium constant of an amidation reaction can give an idea of the relative rate of forward and backward reactions. Not much attention has been given to the equilibrium study of amidation. A paper by Das and co-workers studied the effect of temperature on the equilibrium constant in the reaction of a monoethanolamine with a free fatty acid.^13^ This present research studies the temperature effect on the equilibrium constant for the amidation reaction of diethanolamine and a fatty acid methyl ester.

Moreover, the mass transfer study of the amidation reaction is significant for reactor operation since one of the products, methanol, is volatile and vaporizes from the reacting liquid phase to the surrounding gas phase. Hence, mass transfer effects need to be included in the interpretation of kinetic data in the batch reactor. No prior study has included this, and in this work, we examine this in some detail using a dimensionless mass transfer coefficient parameter. In addition, the amidation reaction is reversible and hence the conversion of this reaction is limited by equilibrium, and thus, requires process manipulation to decrease the rate of the backward reaction. In this reaction, methanol is a byproduct. Therefore, elimination of methanol from the reacting mixture is important to shift the reaction forward. In situ removal of methanol from the reacting mixture in an amidation reactor can improve its conversion.

This process intensification concept, which focuses on *in situ* processes in which, simultaneous separation and reaction take place, requires reduction of the number of processes, resulting in cost saving and reducing the processing time and materials. The process intensification concept of simultaneous byproduct removal is useful, for example, in scaling up and finding the optimum reactor configuration. Examples of applications of separation of the product from the reaction in industry are distillation *in situ*, *e.g.*, the Eastman Kodak process for methyl acetate; membrane reactor for the isomerization of paraffin; extraction *in situ*, *e.g*., the Ruhrchemie–Rhone Poulenc process; and Hofmann reaction of amides.^14−17^

In this research, *in situ* methanol separation is applied. Two stripping process intensification concepts—namely, *in situ* inert gas and vacuum stripping—are introduced to improve the final conversion. These concepts are examined using the mass-transfer-based model. The inert gas stripping model removes byproducts from the liquid phase using an inert gas, while a vacuum pump is applied to the vacuum stripping process. Moreover, the prediction of the vacuum stripping model is also verified with the experimental results, and the potential improvement in the conversion is validated.

The paper is organized in the following manner. Section 2 gives the experimental methods of a closed batch reactor and a batch reactor with vacuum stripping. Section 3 describes mathematical models including the mass transfer and equilibrium models for a closed batch reactor and models for process intensification. All results are discussed in Section 4, and this work is concluded in the last section.

## 2 Experimental Methods

Two amidation experimental systems were set up. One is a closed stirred batch reactor system used for kinetic studies. The other is a stirred batch reactor with vacuum stripping used for testing a concept of process intensification. An anchor impeller with 500 rpm, which has been tested for exhibiting good mixing of the fluids, is used in the batch reactor. Both sets of experiments were carried out at a constant temperature during each experimental run. The temperature of the reactor is controlled with a heater and jacket cooling equipped with a controller. The pressure and temperature are monitored and collected using a data acquisition system. A reactant mixture of 100 mL was charged for a fatty acid methyl ester to diethanolamine at a mole ratio of 1.0:1.06. A sodium methoxide catalyst (CH_3_ONa) solution in diethanolamine at a concentration of 1% w/w is used. The fatty acid methyl ester used in this work contains 10–20 carbon atoms with a distribution that has the average molecular weight of 242.403 g/mol, corresponding to the C_14_ fatty acid. A mild range of temperature—*e.g*., 70–80 °C—was used in this study to limit the occurrence of byproducts, *i.e*., an overcondensate and ester with a free amine. The reactions were carried out in a batch mode with a 500 rpm anchor agitator speed to obtain a homogeneous reaction mixture. The progress of the reaction with time was monitored. The obtained changes of the composition and pressure with time at a constant temperature, which are the important variables in the models, will be used to evaluate the kinetic parameters and to test a process intensification concept. Reactions were separately run for each operating time to avoid sampling, which would have caused a change in the liquid volume. The chemical composition analysis of the fatty acid methyl ester was carried out by high-performance liquid chromatography (HPLC) (Model 6320 ION trap LC/MS). The composition of diethanolamide (AMIDE) was obtained from mass balance calculation. The experiments were repeated for each condition for reliability.

### 2.1 Closed Batch Reactor Setup

**Figure 1:**
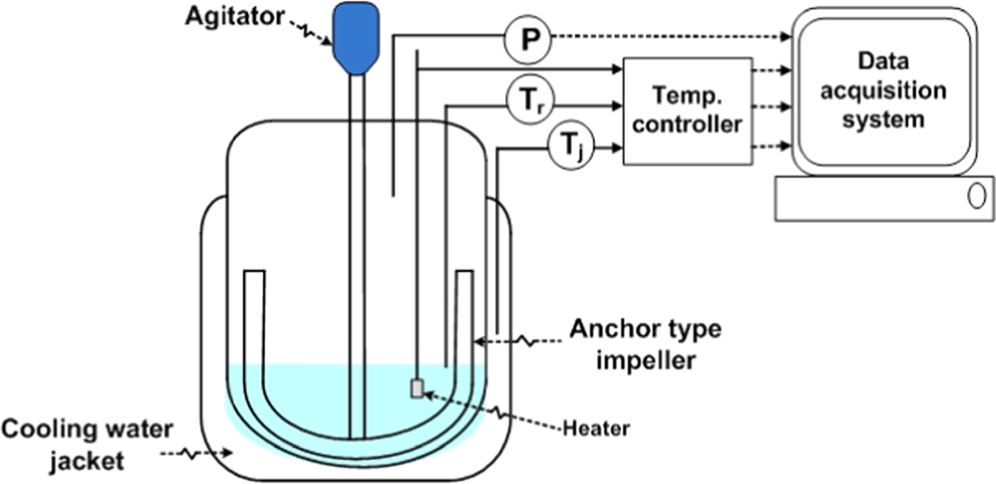
Schematic of a closed batch reactor setup.

A closed batch reactor was experimentally used to study kinetic parameters. The schematic of the experimental setup is shown in Figure 1. A temperature range of 70–80 °C was varied to study the activation energy in the Arrhenius law. The reaction is carried out at a constant temperature for each run. The temperature is continuously monitored during the operating time to ensure that an isothermal condition is obtained during each run. The pressure in a closed batch reactor is initially at 1 bar and increased over time as the reaction proceeds due to an increase of the methanol partial pressure. The progress of the reaction over time was monitored to obtain the rate constant and equilibrium constant. The changes of the concentration and pressure over time were measured until they no longer changed, when the equilibrium was reached. In this work, the experimental results at 480 min were used to study the reaction equilibrium information.

Reaction times of 20, 40, 60, 80, 100, 120, 180, 240, and 480 min were separately run in several small closed batch reactors to eliminate errors in sampling and chemical analysis. The liquid-phase concentrations of components were measured at the above times. The total pressures and temperatures of the reactor are automatically recorded. The liquid level was observed during a batch run, while the liquid volume was measured at the feed and totally discharged at the end. The obtained changes of the composition and pressure with time at a constant temperature, which are the important variables in the models, will be used to evaluate kinetic parameters. The experiments were carried out twice for each temperature. All experimental runs were used for the evaluation of the rate constant for the amidation reaction. Notably, the experimental results at the final time were used to study the reaction equilibrium constants.

### 2.2 Batch Reactor System with Vacuum Stripping

**Figure 2:**
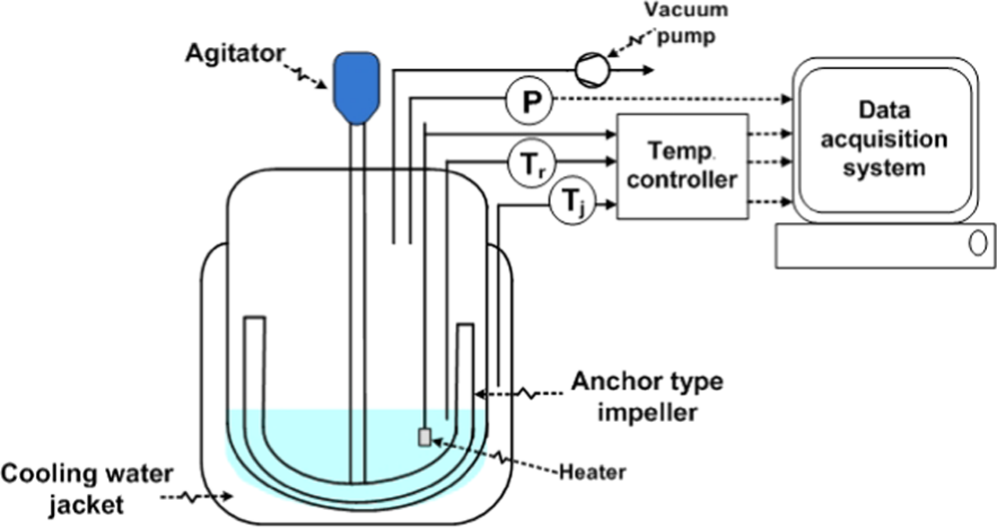
Schematic of a batch reactor with a vacuum pump.

**Figure 3:**
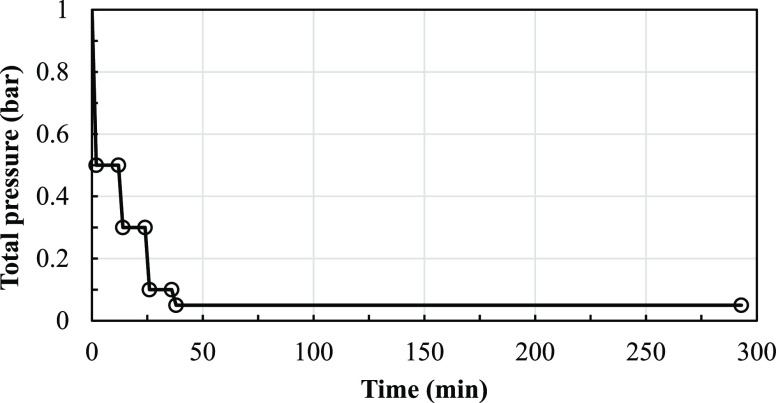
Plot of the imposed stepwise change in the total pressure.

Process intensification with vacuum stripping was carried out in batch stirred-tanks with a vacuum pump. A schematic of an experimental batch reactor with a vacuum pump is shown in Figure 2. A reaction temperature of 80 °C was selected on the basis of it giving the highest conversion of FAME obtained when using a batch system previously. In the vacuum stripping reaction, the pressure was changed stepwise with time, as seen in Figure 3. During the first 2 min, the total pressure was reduced from 1 bar to 500 mbar and held at the latter for 10 min to allow the reacting system to adjust to the new conditions. Then, the pressure was allowed to drop to 300 mbar in 2 min and held constant for 10 min. Next, the pressure was allowed to drop to 100 and 50 mbar—each within 2 min. Under the conditions of 100 mbar, the pressure was also held constant for 10 min. The final total pressure was 50 mbar; this pressure was held until the end. Concentrations of components in the liquid phase were measured at various times along with the change in pressure.

## 3 Mathematical Models

### 3.1 Mass Transfer Model

**Figure 4:**
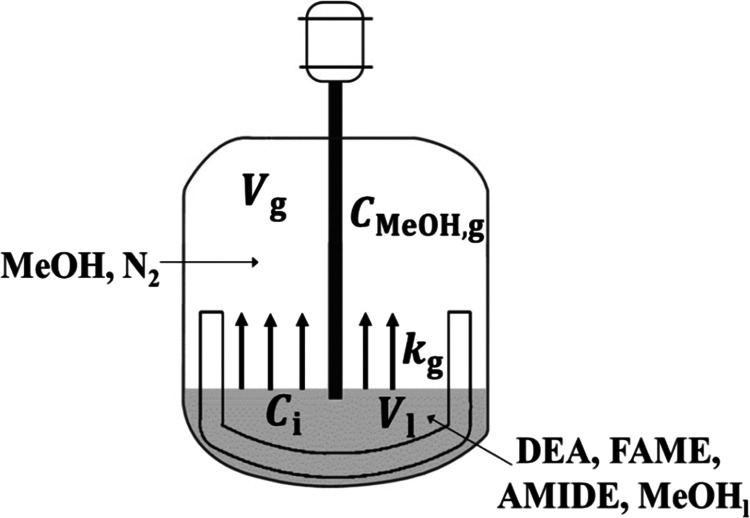
Schematic of a closed batch reactor for modeling.

In this section, the system is modeled as a closed batch reactor, as shown in Figure 4.

In this closed batch reactor, there are two phases, *i.e*., a liquid phase in which the reaction takes place and a gas phase above the liquid phase (with no reaction). A pseudo-homogeneous reaction model is assumed with the first-order reaction for all species, as shown in eq 2
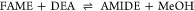
2

The general form of the reaction rate is assumed to be given by the elementary kinetics, as shown in eq 3
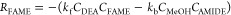
3where *R*_FAME_ is the generation rate of the fatty acid methyl ester (mol/m^3^·min) and *C*_*i*_ is the molar concentration of species *i* in the liquid phase (mol/m^3^).

The modeling assumptions used in this section are as follows:

(a) The liquid mixture is homogeneous.
(b) A pseudo-homogeneous reaction model is assumed with the first-order reaction for all species.
(c) Liquid and vapor are well mixed in each phase.
(d) The reactor is isothermal, and the gas and liquid are in thermal equilibrium.
(e) The liquid volume is assumed to be constant during the reaction.
(f) DEA, FAME, and AMIDE are nonvolatile. Only methanol is volatile.
(g) The reaction occurs only in the liquid phase.

DEA, FAME, and AMIDE are nonvolatile and stay in the mixture without evaporation due to their high boiling points, making methanol the only volatile component. The liquid volume is assumed to be constant during the reaction. Although the vaporization of methanol can reduce the liquid volume, the loss of the liquid volume due to methanol vaporization in this work is small, less than 0.63%. The assumption of a well-mixed liquid in a stirred reactor can be applied in this work since a small volume of the liquid with a high speed of the agitator is used. If the model is applied to a large-scale reactor, a mixing parameter indicating a nonideal flow should be included in the model.

The mass transfer models for a small closed batch reactor can be written for liquid and gas phases as follows.

All nonvolatile species mass balances in the liquid phase for a closed batch reactor are shown in eqs 4–6
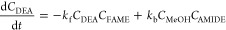
4
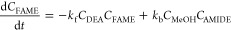
5
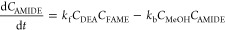
6

During the amidation reaction, methanol vaporizes from the liquid-phase mixture and enters the gas phase above the reacting liquid. Hence, the mass balance of methanol in the liquid phase should include a term for the mass transfer rate of methanol from the liquid to gas phases, as shown below
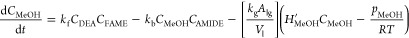
7where *k*_g_ is the coefficient of the mass transfer from the liquid to the gas phase (m/min), *A*_lg_ is the gas–liquid interfacial area for mass transfer (m^2^), *k*_f_ and *k*_b_ are the forward and backward reaction rate constants, respectively (m^3^/mol·min), *p*_MeOH_ is the partial pressure of methanol in the gas phase (bar)*, t* is the reaction time (min)*, V*_l_ is the volume of the liquid phase (mL), *R* is the gas constant (L·bar/mol·K), *T* is the temperature (K), *H*_MeOH_^′^ is the dimensionless partition constant, and the last term in eq 7 is the mass transfer rate of volatile methanol from the liquid to the gas phase. Note that at the vapor–liquid equilibrium conditions, *C*_MeOH,g_ = *H*_MeOH_^′^*C*_MeOH_.

Initial conditions for solving the transient mass balances of the liquid phase are
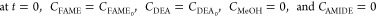


In addition, the total pressure in the closed batch reactor consists of nitrogen and methanol partial pressures in the gas phase. Initially, the gas phase comprises nitrogen only. After the reaction occurs, methanol vaporizes into the gas phase. The partial pressure of methanol in the reactor increases with time, leading to an increase in the total pressure, while the partial pressure of nitrogen remains constant. Moreover, the gas phase itself is assumed to be well mixed, and the ideal gas law is applied. Performing a mass balance on the gas phase requires including a term for methanol transfer
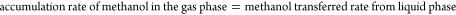
8
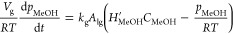
9where *V*_g_ is the volume of the gas phase (mL). The initial condition for solving the transient mass balance on the gas phase is given below
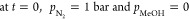


The value of the coefficient of mass transfer is needed for a detailed simulation. It is easier to examine the effect of the mass transfer parameter using dimensionless versions of governing equations. The details of this will be shown in the next section

### 3.2 Dimensionless Closed Batch Reactor Model with Mass Transfer Effects

In this section, the sensitivity of the model to the mass transfer coefficient is examined in terms of the dimensionless mass transfer coefficient (α_lg_). It is easier to examine the effect of this parameter using a dimensionless setting of governing equations. All concentrations are scaled by a reference concentration, *C*_ref_, which here is taken as the initial FAME concentration. Meanwhile, time is scaled as t* = *k*_f_*C*_ref_*t*. This leads to the following set of equations.

For DEA, FAME, and AMIDE species, the mass balance for the liquid phase is
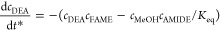
10
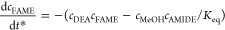
11
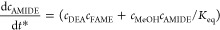
12where *c*_*i*_ is the dimensionless molar concentration of species *i* in the liquid phase, namely, *C*_*i*_/*C*_ref_.

The equilibrium constant, *K*_eq_, is expressed as
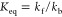
13

For methanol species, the mass balance for the liquid phase is
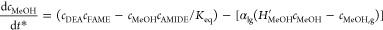
14

The dimensionless mass transfer parameter, α_lg_, is given as
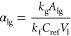


When the liquid volume is constant, *V*_l_ = *V*_l_in__, where *V*_l_in__ is the initial liquid volume (m^3^). Therefore, α_lg_ can also be defined as
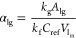
15

This latter definition will be used later in the section of stripping systems.

The mass balance for methanol species in the gas phase is written as
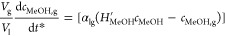
16where *c*_MeOH,g_ denotes the dimensionless molar concentration of methanol in the gas phase.

These equations (eqs 10–16) are used to examine the sensitivity of the overall model to the dimensionless mass transfer coefficient (α_lg_). As α_lg_ is very high, the above mass transfer models are remodeled to form equilibrium models, which will be discussed in the next section.

### 3.3 Equilibrium Model for the Closed Batch Reactor

In the equilibrium model, it is expected that the gas and the liquid phase will be in equilibrium, and consequently, a simpler equilibrium model can be used. In such a case, eqs 7 and 9 are combined together to eliminate the mass transfer terms. A single mass balance for methanol in gas and liquid phases, for which the equilibrium condition is substituted, is thereby obtained
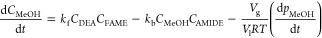
17

For the equilibrium model, the partial pressure of methanol in the bulk gas, *p*_MeOH_, attains the same value as the partial pressure of methanol at the interface, *p*_MeOH_^*^. Therefore, eq 17 becomes
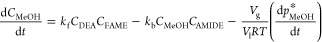
18

The partial pressure of methanol at the interface is defined using Raoult’s law of vapor and liquid equilibrium, as shown in eq 19. An activity coefficient, γ_MeOH_, is included to take care of the nonideality of the liquid phase^18^
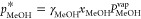
19where γ_MeOH_ is the liquid activity coefficient of methanol, *p*_MeOH_^*^ is the partial pressure of methanol at the interface (Pa), *P*_MeOH_^vap^ is the vapor pressure of pure methanol (Pa), and *x*_MeOH_ is the mole fraction of methanol in the liquid phase, which in fact is equal to *N*_MeOH_/*N*_tot_.
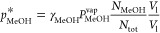
20where *N*_MeOH_ is the number of moles of methanol in the liquid phase (mol) and *N*_tot_ is the total moles of the liquid mixture (mol).

In this way, the partition constant in terms of the above system parameters is defined as
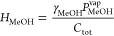
21where *C*_tot_ is simply the total concentration in the liquid, namely, *N*_tot_/*V*_l_ and *H*_MeOH_ are the partition constants (Pa·m^3^/mol). Note that the partition constant for the gas–liquid equilibrium (*H*_MeOH_) is related to the dimensionless partition constant *H*_MeOH_ as *H*_MeOH_^′^ = *H*_MeOH_/*RT*.

Thus, at equilibrium, eq 20 becomes
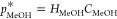
22

The vapor pressure of methanol (*P*_MeOH_^vap^) can be calculated from the Antoine equation
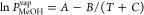
23with the values for the constants^19^

*A* = 23.402; *B* = 3593.4; and *C* = −34.92 with all SI units, *i.e*., *T* in K and pressure in Pa.

Initial conditions for solving the transient mass balance on the gas phase (eq 18) are
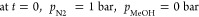


The concentration of methanol in the liquid phase (*C*_MeOH_) in the mass balance equations is determined by the equilibrium expression, eq 22. The mass balance, eq 18, for methanol thereby becomes
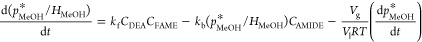
24

The measured partial pressure of methanol and the concentration of the fatty acid methyl ester at various times are fitted to the equilibrium models; consequently, the rate constants (*k*_f_, *k*_b_) and the activity coefficient can be obtained.

### 3.4 Models for Process-Intensification Concepts

The amidation reaction is reversible, and hence the conversion of this reaction is limited by the equilibrium. Accordingly, a method to decrease the rate of the reversible reaction is required. One such method—indeed the adopted one—revolves around the byproducts. In this reaction, one of the products is volatile, *i.e*., methanol, which is a byproduct. Hence, elimination of methanol from the reacting mixture is important to shift the reaction forward. Two process-intensification concepts of simultaneous volatile product removal are demonstrated to increase the final conversion, especially when one wants to scale up and arrive at an optimum reactor configuration. Two ways to achieve the volatile product removal are examined here: (i) the flowing additional inert gas into the system so as to remove the volatile species and (ii) vacuum stripping whereby the operating pressure is progressively lowered.

#### 3.4.1 Process Intensification by Inert Gas Stripping

**Figure 5:**
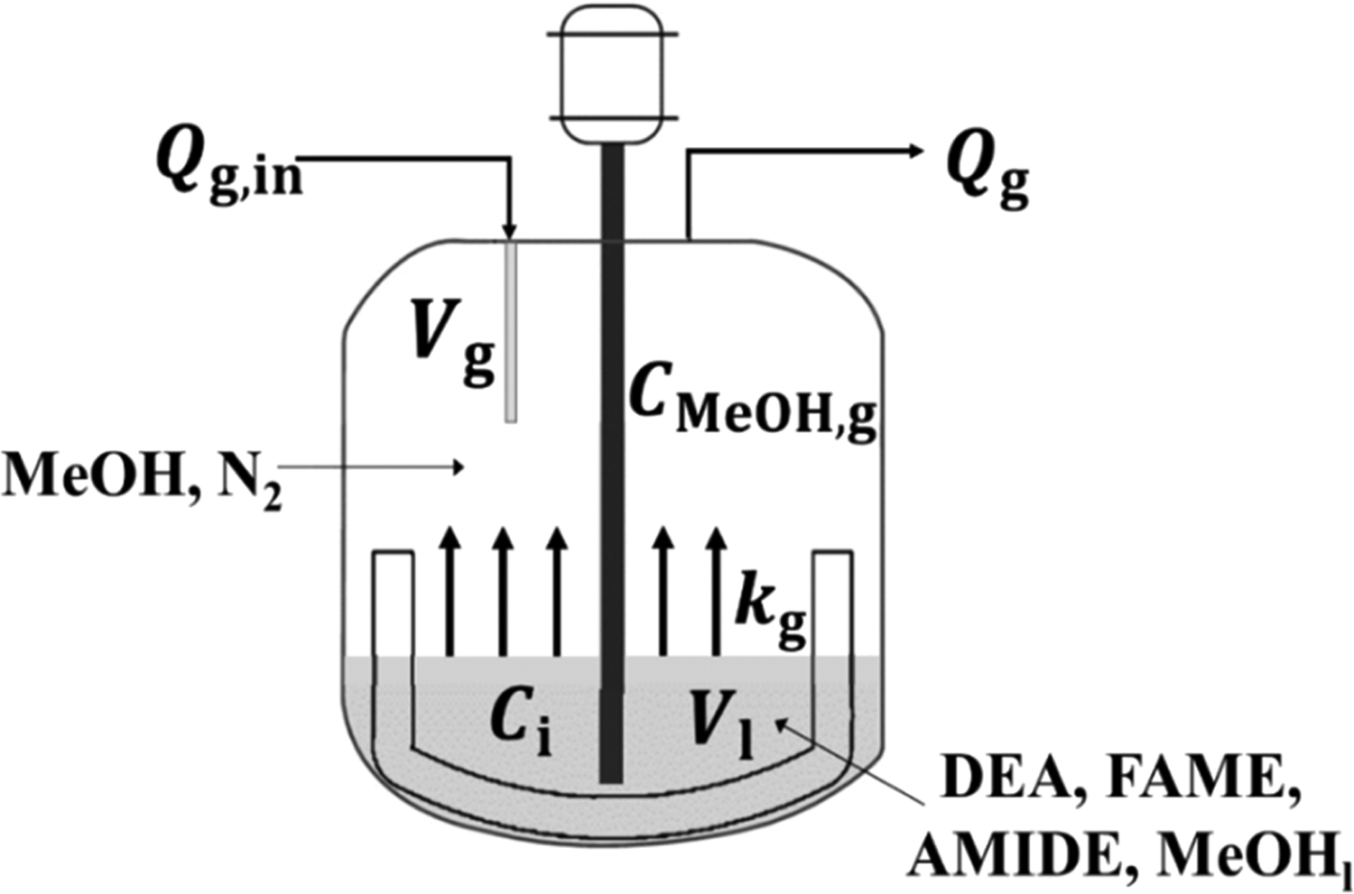
Schematic of a batch reactor with inert gas stripping for modeling.

Inert gas stripping is applied in a process-intensification concept by flowing an additional inert gas into the system to remove the volatile methanol. Figure 5 shows the process-intensification arrangement of inert gas stripping for modeling. The system consists of a batch reactor with inert gas stripping. The analysis of the inert gas stripping model is similar to that provided in the book of Ramachandran.^20^ This system is often nicknamed as a reactor–separator combo (combination) system. The idea of this method is to make the conditions favorable for methanol in the liquid phase to transfer to the gas phase. To this end, the key dimensionless parameter is identified, and the efficiency of gas stripping is examined in this section.

All species of DEA, FAME, and AMIDE are nonvolatile and remain in the mixture without evaporation. An isothermal condition of a small reactor is assumed and the gas and liquid are in thermal equilibrium. The pressure in a reactor with inert gas stripping is initially at 1 bar and changes over time as the reaction proceeds due to methanol vaporization.

The mass balance model equations in dimensionless forms for DEA, FAME, and AMIDE in the liquid phase for the current stripping setup are shown in eqs 25–27. Note that the liquid volume is changed over time. The change of the liquid volume is included in the model.

For DEA, FAME, and AMIDE species, the mass balance for the liquid phase is
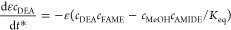
25
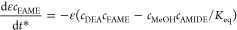
26
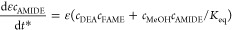
27where ε is the liquid volume per initial liquid volume.
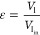


The mass balance of methanol in the liquid phase is described in eq 28.
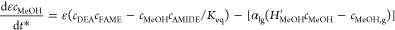
28where
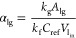


However, methanol is a volatile component. The dimensionless material balance in the gas phase is augmented to include flow removal for this gas stripping system as shown in eq 29.
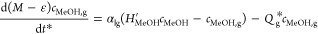
29where *M* = total reactor volume/initial volume of the liquid mixture  and*V*_R_ is the total reactor volume.

Note that the mass balance in eq 29 assumes that the gas phase is well mixed in the system. The last term is the methanol removal rate due to gas stripping, in which *Q*_g_^*^ is the dimensionless volumetric flow rate of the stripping gas. *Q*_g_^*^ characterizes the effect due to simultaneous gas removal, which is the ratio of the reaction time to the gas residence time based on the liquid volume in the reactor, defined as eq 30.
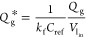
30where *Q*_g_ is the volumetric flow rate of the stripping gas (m^3^/min). The reaction time is obtained from 1/*k*_f_*C*_ref_. The gas residence time ((*V*_l_in__)/*Q*_g_) should be of the same order as the reaction time to provide enough time for product stripping.

The process intensification of gas stripping is examined using a large mass transfer parameter (α_lg_ = 4), which provides the highest potential for process intensification. At a large value of the mass transfer parameter (α_lg_), a condition of equilibrium between the methanol gas and liquid is reached (*c*_MeOH,g_ = *H*_MeOH_^′^*c*_MeOH_). In such a case, eqs 28 and 29 are combined together for eliminating the mass transfer term, the same way as in Section 3.3.

The difference from Section 3.3 is that the dimensionless mass balance in the gas phase includes the term of flow removal. This leads to the following mass balance equation for methanol in the liquid phase
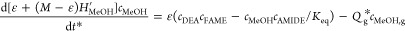
31

The change of liquid volume over time is obtained from the mass balance, as shown in eq 32.
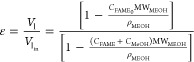
32where MW_MEOH_ is the molecular weight of methanol (kg/mol) and ρ_MEOH_ is the liquid density of methanol (kg/m^3^).

The above equations (eqs 25–27 and 31) are used to examine the effect of the gas flow rate on the conversion of FAME. The obtained FAME conversion for the stripping case will be compared to that for the base case of no gas flow.

#### 3.4.2 Process Intensification by Vacuum Stripping

**Figure 6:**
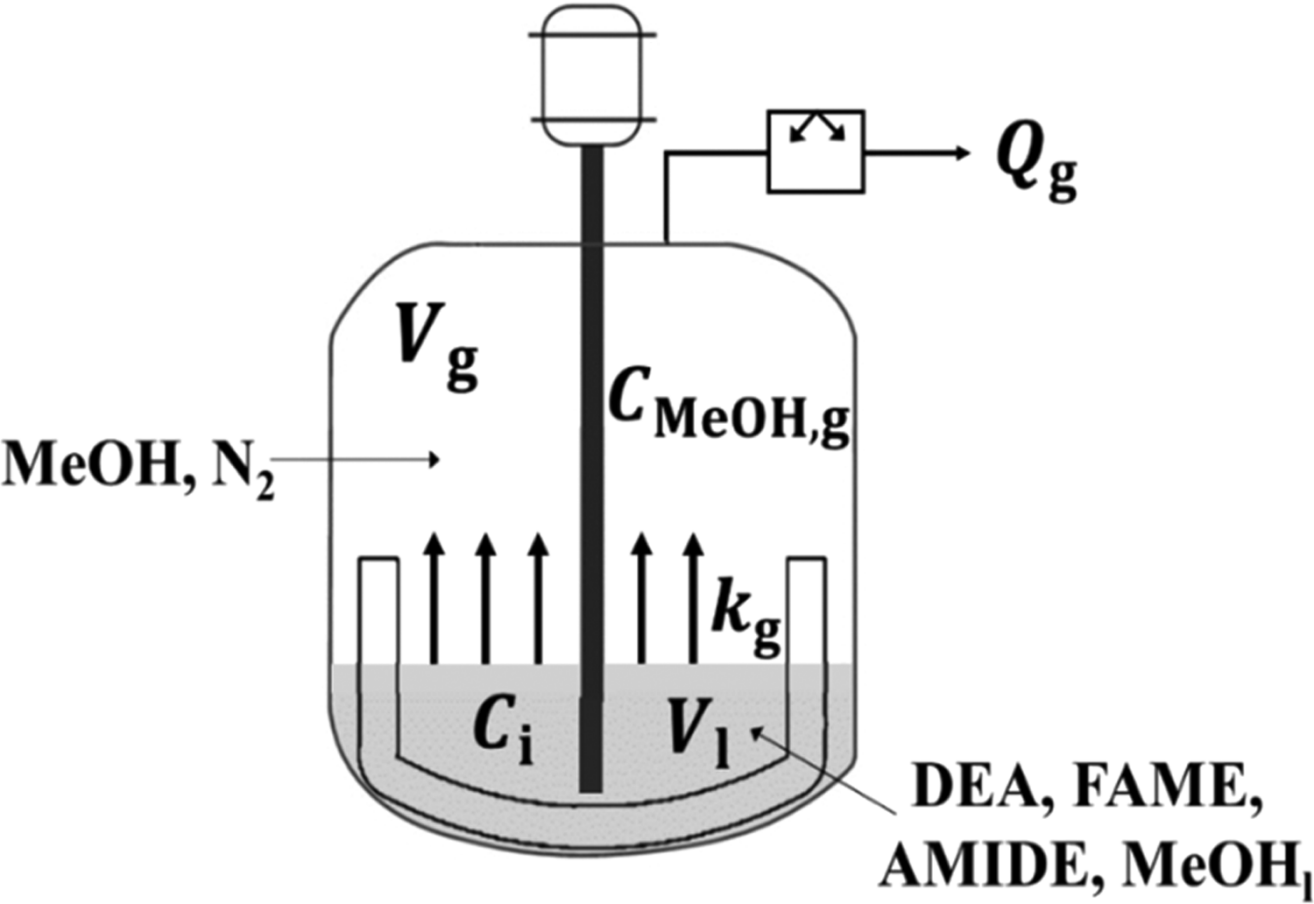
Schematic of a batch reactor with vacuum stripping for modeling.

Another way to simultaneously remove the volatile methanol from the reacting system to shift the equilibrium conversion is vacuum stripping. This can be conveniently done using a vacuum pump to remove the gas mixture above the liquid. A batch reactor system with vacuum stripping for modeling is shown in Figure 6.

An isothermal condition of a small reactor is assumed and the gas and liquid are in thermal equilibrium. The liquid mass balances of DEA, FAME, AMIDE, and MeOH for this system with vacuum stripping are shown in eqs 33–36. Note that the change in the liquid volume is included in the models. For the gas phase, however, the batch reactor with vacuum stripping needs additional mass balances—both for nitrogen and methanol. The general form of species mass balances in the gas phase is given below
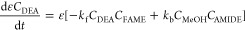
33
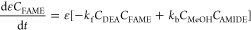
34
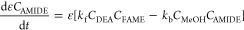
35
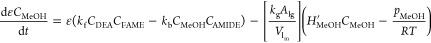
36
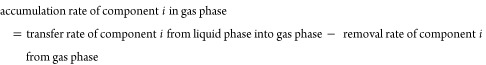
37

In this system, no nitrogen is transferred from the liquid phase. Thus, the mass balance of nitrogen is
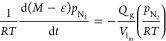
38

Initially, the nitrogen pressure is 1 bar.

Meanwhile, throughout the process, methanol continually vaporizes from liquid to gas phases. The mass balance of methanol in the gas phase in terms of the methanol partial pressure is presented in eq 39
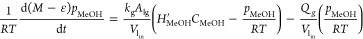
39

This mass balance of methanol for the vacuum system (eq 39) is similar to that for the stripping system (eq 29), except that the former is written in terms of pressure and the latter is written in terms of concentration.

Since there are only two gaseous species, the rate of change of the total pressure is
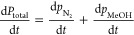
40

These two equations, eqs 39 and 40, together with eqs 33–36 and the plot of vacuum pressure over time (Figure 3) can be used to examine a method of vacuum stripping.

In this study, the pressure was reduced stepwise with time using a vacuum pump. Figure 3 shows the pressure plot as a function of time required for this work. This change of pressure over time can be experimentally obtained. The pressure remains a piecewise constant (d*P*/d*t* is zero) for each time interval. The exit volumetric flow rate of gas removal (*Q*_g_) in the calculation is adjusted to satisfy the pressure profile.

The required pressure drops in vacuum stripping showing that short time intervals over the pressure must drop from one level to the next level. The pressure is known in the differential equations (eqs 39 and 40), while *Q*_g_ is unknown. Over a fixed time interval, *Q*_g_ is obtained through optimization of eqs 39 and 40 to match the required pressure. In addition to eqs 39 and 40, eqs 33–36 are required. These four equations are used for solving *C*_MeOH_, which is a variable in eq 39.

## 4 Results and Discussion

### 4.1 Effect of Mass Transfer and the Equilibrium Approach

The sensitivity of the model to the dimensionless mass transfer parameter (α_lg_) was investigated. The mass transfer models (eqs 10–12, 14, and 16) are used to test the effect of mass transfer. The parameters *k*_f_, *k*_eq_, and γ_MeOH_ shown in the models are constant at a constant temperature. The rate constants are considered as intrinsic rate constants since the mass transfer effect is separately considered in the model. These three parameters were obtained from fitting of experimental data of a closed batch reactor to equilibrium models. The details of assessing these parameters will be shown in the next section (Section 4.2).

**Figure 7:**
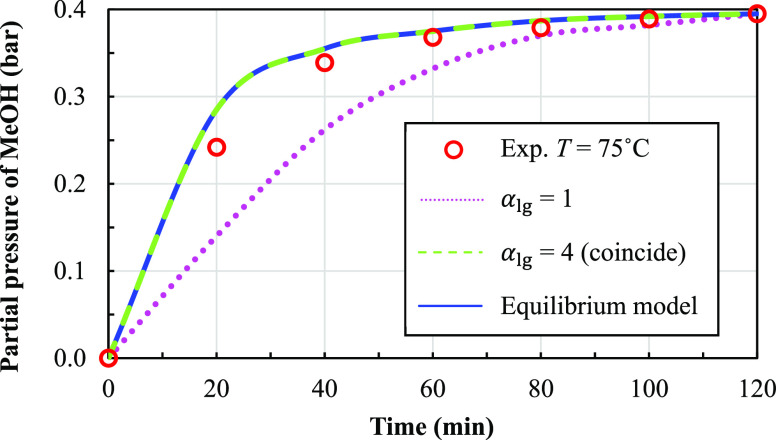
Change in the methanol partial pressure with time at a reaction temperature of 75 °C. The dotted line is for α_lg_ = 1 and dot markers indicate experimental results. The dark solid line is the equilibrium model, with which the results for α_lg_ = 4 coincide.

The sensitivity of the overall model to the dimensionless mass transfer coefficient (α_lg_) is studied by varying α_lg_ while keeping the other parameters constant. The operating conditions and parameters used in this study are shown in Table 1. The change of methanol partial pressure for various α_lg_ parameters at 75 °C is shown in Figure 7. It can be seen that the methanol partial pressure progressively increases with the mass transfer parameter due to more methanol transferring from the liquid into the bulk gas. Finally, the model approaches the equilibrium value for α_lg_ = 4. Figure 7 shows that the results for α_lg_ = 4 coincide with the results of the equilibrium model. In addition, it was observed that the results for α_lg_ = 4 and for the equilibrium model are in good agreement with the experimental results. Thus, the equilibrium model can be applied along with the experimental data to evaluate the kinetic and equilibrium parameters, which will be shown in the next section. Note that the details of the experimental data and calculation results of the equilibrium model in this figure will be discussed in the following section.

**Table 1:** Operating Conditions and Parameters Used in the Study of the Mass Transfer Effect

initial FAME concentration (mol/m^3^)	2509
initial DEA concentration (mol/m^3^)	2650
volume of the gas (mL)	900
volume of the liquid mixture (mL)	100
temperature (°C)	75
*K*_eq_ at 75 °C (obtained from Section 4.2)	16.44
*k*_f_ at 75 °C (m^3^/mol·min) (obtained from Section 4.2)	2.71 × 10^–5^
γ_MeOH_ at 75 °C (obtained from Section 4.2)	0.7
*C*_ref_, (mol/m^3^) = initial FAME concentration (mol/m^3^)	2509
dimensionless mass transfer coefficient (α_lg_)	1, 4

### 4.2 Evaluation of Kinetic and Equilibrium Parameters

The kinetic and equilibrium parameters of the amidation reaction can be obtained from fitting the experimental data of a closed batch reactor with the equilibrium models.

#### 4.2.1 Experimental Results

This section shows the experimental results of amidation in a closed batch reactor. The operating conditions of experiments in the closed batch reactor are shown in Table 2. The experiments were carried out twice for each temperature.

**Table 2:** Operating Conditions Used in Experiments of the Closed Batch Reactor

initial pressure (bar)	1
initial methanol partial pressure (bar)	0
initial FAME concentration (mol/m^3^)	2509
initial DEA concentration (mol/m^3^)	2650
volume of the gas (mL)	900
volume of the liquid mixture (mL)	100
agitator speed (rpm)	500
temperature (°C)	70, 75, 80
*k*_f_, *k*_eq_, and γ_MeOH_	variables are calculated

**Figure 8:**
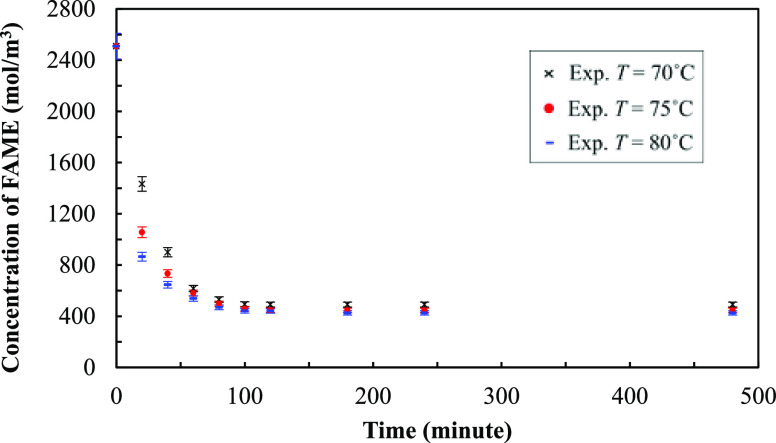
Experimental concentrations of FAME over time at different temperature conditions.

**Figure 9:**
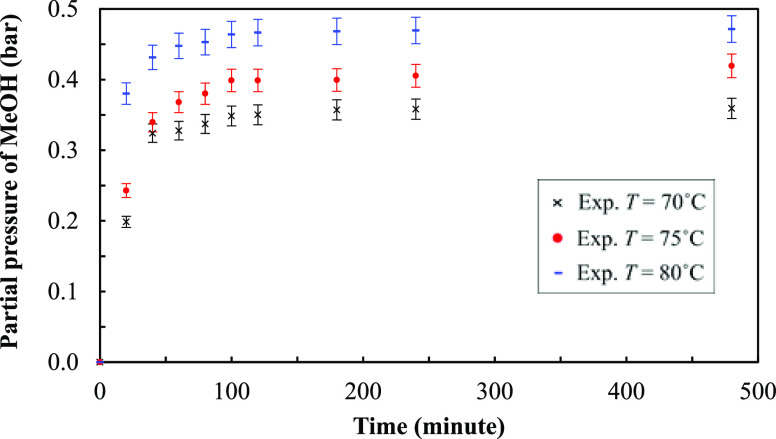
Experimental partial pressures of methanol over time in different temperature conditions.

The measured results are shown in terms of changes in the total partial pressure and concentration of the fatty acid methyl ester over time. Mean concentrations of FAME and the error bars at 70, 75, and 80 °C are shown in Figure 8. The methanol partial pressure at each operating time can be calculated from the increase of the total pressure over time. Figure 9 shows the mean of methanol partial pressures and the error bars at different temperature conditions. The maximum error bars of both data sets are ±4%.

Figure 9 shows that for all temperature conditions, the partial pressure of methanol increases sharply in the interval from 0 to 20 min and then increases only slightly from 40 to 100 min. After this, until a time of 120 min, the partial pressure of methanol is almost constant. The partial pressure of methanol increases with temperature because a high reaction rate leads to more methanol being vaporized. Figure 8 shows that the experimental concentration of the fatty acid methyl ester decreases sharply in the initial period of the reaction and then slightly decreases until the concentration becomes constant at the end. Moreover, at high temperatures, more conversion of FAME is observed due to a higher reaction rate. The final conversions at 480 min and at the temperatures of 70, 75, and 80 °C were found to be 80.45, 82.46, and 83%, respectively.

#### 4.2.2 Evaluation of Kinetic and Equilibrium Parameters

**Figure 10:**
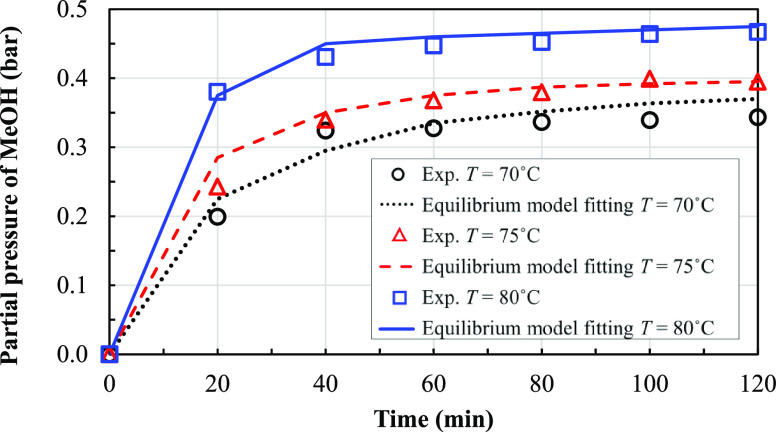
Obtained fitting of the experimental change of methanol partial pressure over time with the equilibrium model for different temperature conditions.

**Figure 11:**
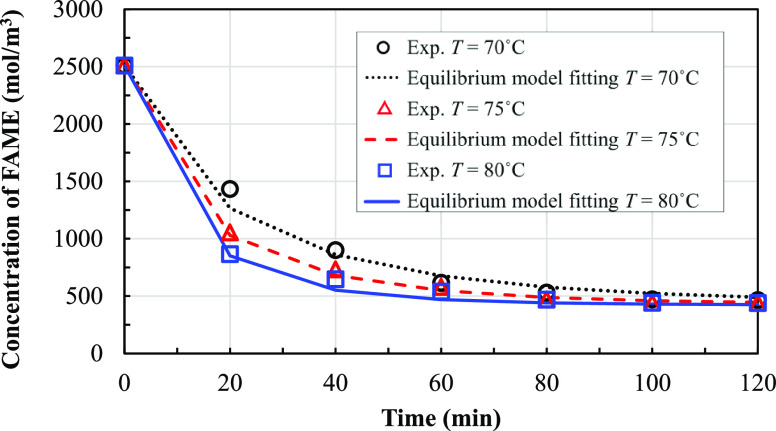
Obtained fitting of the experimental change of FAME concentration over time with the equilibrium model for different temperature conditions.

The kinetic and equilibrium parameters of the amidation reaction can be obtained from fitting the experimental partial pressure of methanol and the concentration of the fatty acid methyl ester to the equilibrium model. The equilibrium model consists of four main mass balance equations including three mass balances of three components in the liquid phase (eqs 4–6) and a single mass balance for methanol in gas and liquid phases (eq 24). Fitting the experimental partial pressure of methanol and concentration of FAME over time to these four equations, three parameters—*k*_f_, *K*_eq_, and *H*_MeOH_ can be assessed. The corresponding activity coefficient can then be calculated from *H*_MeOH_ using eq 21. In fitting, solving these four equations with the initial conditions shown in Table 1, the concentration and pressure can be calculated at each time until the end of the experiments. The experimental data at the final time (at 4 h) is used to roughly fit for the reaction equilibrium constant. Then, all experimental data over time are used to get the best fits for the forward rate constant, reaction equilibrium constant, and partition constant. Minimizing the sum of squared error is considered to get the best fit. All parameters obtained for the three temperature conditions are shown in Table 3. Note that the backward rate constant (*k*_b_) is obtained using eq 13 for calculation. Figures 10 and 11 show the obtained fitting curves for the methanol partial pressure and FAME concentration over time, respectively.

**Table 3:** Rate Constants, Equilibrium Constants, Partition Constants, and the Liquid Activity Coefficient Obtained from Fitting

temperature (°C)	*K*_eq_	*k*_f_ (m^3^/mol·min)	*k*_b_ (m^3^/mol·min)	*H*_MeOH_ (Pa·m^3^/mol)	γ_MeOH_
70	15.95	1.84 × 10^–5^	1.15 × 10^–6^	19.531	0.8
75	16.44	2.71 × 10^–5^	1.65 × 10^–6^	20.585	0.7
80	16.92	3.95 × 10^–5^	2.33 × 10^–6^	24.651	0.7

The results show that the forward reaction rate constant (*k*_f_) increases with temperature. Moreover, the backward reaction rate constant (*k*_b_) is much lower than *k*_f_. Therefore, the rate of the backward reaction is not significant for amidation but is still necessary to limit the complete conversion of reactants.

**Figure 12:**
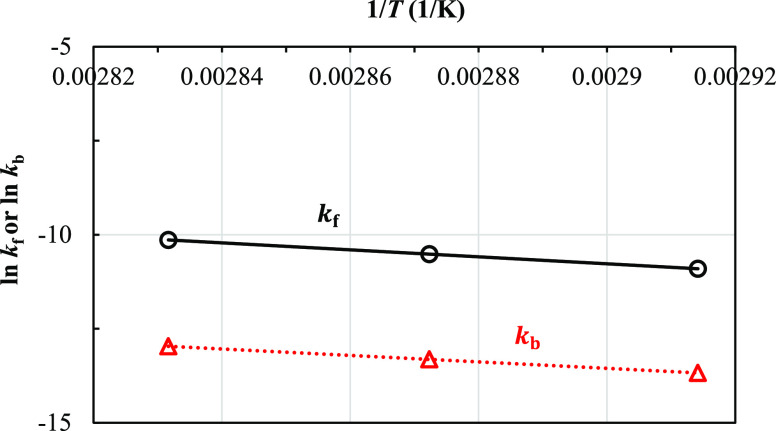
Plots of ln *k*_f_*versus* 1/*T* and ln *k*_b_*versus* 1/*T* for finding activation energy values of the forward and backward reactions.

The effects of temperature on rate constants are described by Arrhenius’ law. Figure 12 shows plots of ln *k*_f_*versus* 1/*T* and ln *k*_b_*versus* 1/*T* for finding activation energy values of the forward and backward reactions, respectively. The activation energy values for forward and backward reactions (*E*_f_ and *E*_b_) obtained from the slopes of the graphs were found to be 77.12 and 71.17 kJ/mol, respectively. Therefore, the rate constants of forward and backward reactions can be written as a function of temperature by Arrhenius’ law as follows
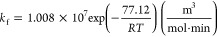
41
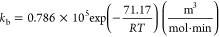
42

**Figure 13:**
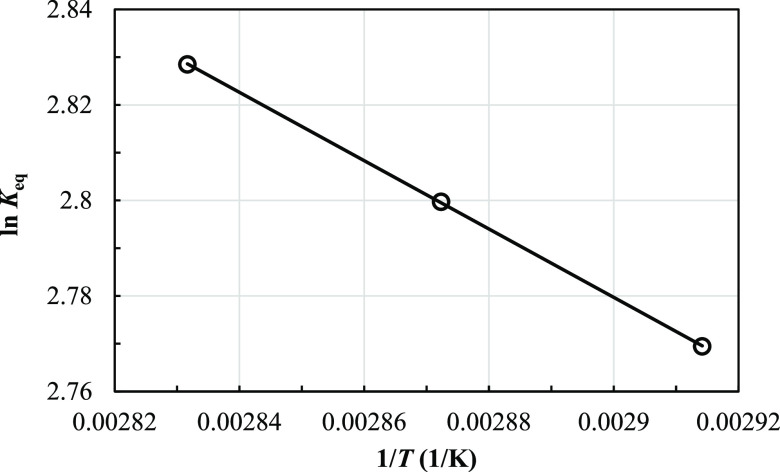
Plot of 1/*K*_eq_ and 1/*T* for finding the enthalpy of reaction.

The effect of temperature on the equilibrium constant is shown in Table 3. It was found that the equilibrium constants increased slightly with temperature, indicating a mild endothermicity. Such an effect of mild endothermicity has also been found in the available research on other types of amidation.^13^ The effect of temperature on the equilibrium constant can be explained by the van’t Hoff equation ., where Δ*H*_rxn_ is the enthalpy of reaction (kJ/mol). The plot of ln *K*_eq_ and 1/*T*, as shown in Figure 13, gives the enthalpy of this amidation reaction—namely, 5.95 kJ/mol. In addition, the enthalpy of reaction can also be calculated from the fundamental thermodynamic equation. The enthalpy of reaction is the difference of activation energy values of the forward reaction and backward reaction (Δ*H*_rxn_ = *E*_f_ – *E*_b_). Thus, the enthalpy of the amidation reaction calculated from the activation energy is 5.95, which corresponds to the value obtained from the slope of Figure 13. In addition, the liquid activity coefficient is slightly affected by temperature and it is in the range of 0.7–0.8 for the temperature of 70–80 °C.

#### 4.2.3 Prediction of Parameters from Thermodynamics

Apart from experimental data on transients toward the reaction equilibrium, the parameters can be predicted from thermodynamic information.

(a) Reaction equilibrium constant
  The reaction equilibrium constant can be estimated from Gibbs free energy as the following equation
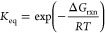
43where Δ*G*_rxn_ is the Gibbs free enthalpy change of reaction (kJ/mol), which is obtained from the free enthalpy of formation of component *i*, *G*_f,*i*_
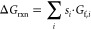
44where *s*_*i*_ is the stoichiometric number of species *i* and *G*_f,*i*_ is the Gibbs free enthalpy of formation of species *i* (kJ/mol).
(b) Reaction enthalpy and activation energy
  The reaction enthalpy can be calculated from the activation energy as follows
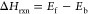
45where Δ*H*_rxn_ is the reaction enthalpy (kJ/mol) and *E*_f_ and *E*_b_ are the activation energies of the forward and backward reactions (kJ/mol), respectively. In addition, the reaction enthalpy can be calculated from the enthalpy of formation of component *i* as follows
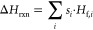
46where *H*_f,*i*_ is the enthalpy of formation of component *i* (kJ/mol).
(c) Activity coefficient of the liquid phase,

The activity coefficient of the liquid phase (γ_MeOH_) can be predicted by the UNIFAC method.

The enthalpy of reaction and Gibbs free energy change of the amidation reaction are calculated using thermodynamic information. In this work, the enthalpy of formation and Gibbs free energy of each component in the amidation reaction were simply estimated by the Joback method, which is a group-contribution method. The average enthalpy of the amidation reaction calculated for the temperature range of 70–80 °C is shown in Table 4. In addition, the reaction equilibrium constant calculated from the Gibbs free energy change at 75 °C is shown in Table 4. The comparisons of equilibrium constants and the enthalpy of reaction obtained from thermodynamic information and experiments are also shown in Table 4.

**Table 4:** Comparison of Equilibrium Constants and the Enthalpy of Reaction from Thermodynamic Information and Experiments at 75 °C

parameter	experiments	thermodynamics
enthalpy of reaction (kJ/mol) for 70–80 °C	5.95 (endothermic)	–6.90 (exothermic)
equilibrium constant at 75 °C	16.44	38.70
liquid activity coefficient of methanol at 75 °C	0.7	0.7

The enthalpy of reaction obtained from calculations and experiments shows that the reaction is not far from isothermal conditions although they give an opposite nonisothermal behavior. The absolute error is 12.85 kJ/mol. The discrepancies of both values may come from errors in modeling of chemical structures of the fatty acid methyl ester and fatty acid diethanolamide, which are long-chain compounds and derivatives of natural compounds. The natural compounds contain distributions of carbon atoms. The liquid activity coefficient of methanol was estimated using the UNIFAC method. The calculation liquid activity coefficient of methanol (γ_MeOH_) at 75 °C was found to be 0.7. The activity obtained from fitting with experimental data matches the one obtained from thermodynamic information very well.

### 4.3 Result of Process Intensification by Inert Gas Stripping

Process intensification by inert gas stripping is focused on in this section. As described in Section 3.4.1, inert gas stripping is used to increase the removal of methanol from the liquid phase. In this section, the effect of the stripping gas flow rate in terms of the dimensionless parameter *Q*_g_^*^ is studied. *Q*_g_^*^ is the ratio of the reaction time to the gas residence time based on the liquid volume in the reactor. Solving the inert gas stripping model (eqs 25–27 and 31) with the operating conditions shown in Table 5, the conversions of the fatty acid methyl ester were obtained. The rate constant, equilibrium constant, and the liquid activity used in the calculation are obtained from Section 4.2

**Table 5:** Operating Conditions Used in the Performance Calculation for the Reactor with Inert Gas Stripping

initial FAME concentration (mol/m^3^)	2509
initial DEA concentration (mol/m^3^)	2650
initial volume of the gas (mL)	900
initial volume of the liquid mixture (mL)	100
*M* = total reactor volume/initial volume of liquid mixture	10
temperature (°C)	80
*K*_eq_ at 80 °C (obtained from Section 4.2)	16.92
*k*_f_ at 80 °C (m^3^/mol·min) (obtained from Section 4.2)	3.95 × 10^–5^
γ_MeOH_ at 80 °C (obtained from Section 4.2)	0.7
*C*_ref_, (mol/m^3^) = initial FAME concentration (mol/m^3^)	2509
*Q*_g_, (m^3^/min) (*Q*_g_^*^ = 10)	9.91 × 10^–5^

For the present case of a measured temperature of 80 °C, the reaction time (*i.e*., 1/*k*_f_*C*_ref_) is of the order of 14.7 min. The gas residence time (*i.e*., *V*_l_in__/*Q*_g_) should be at least the same order of magnitude or higher than the reaction time to provide enough time for product stripping. *Q*_g_^*^ of 10 is used for this study to ensure enough time for stripping. Thus, the corresponding gas stripping gas flow rate (*Q*_g_) for this case is 9.91 × 10^–5^ m^3^/min, which is within the usual operating range of gas–liquid systems.

**Figure 14:**
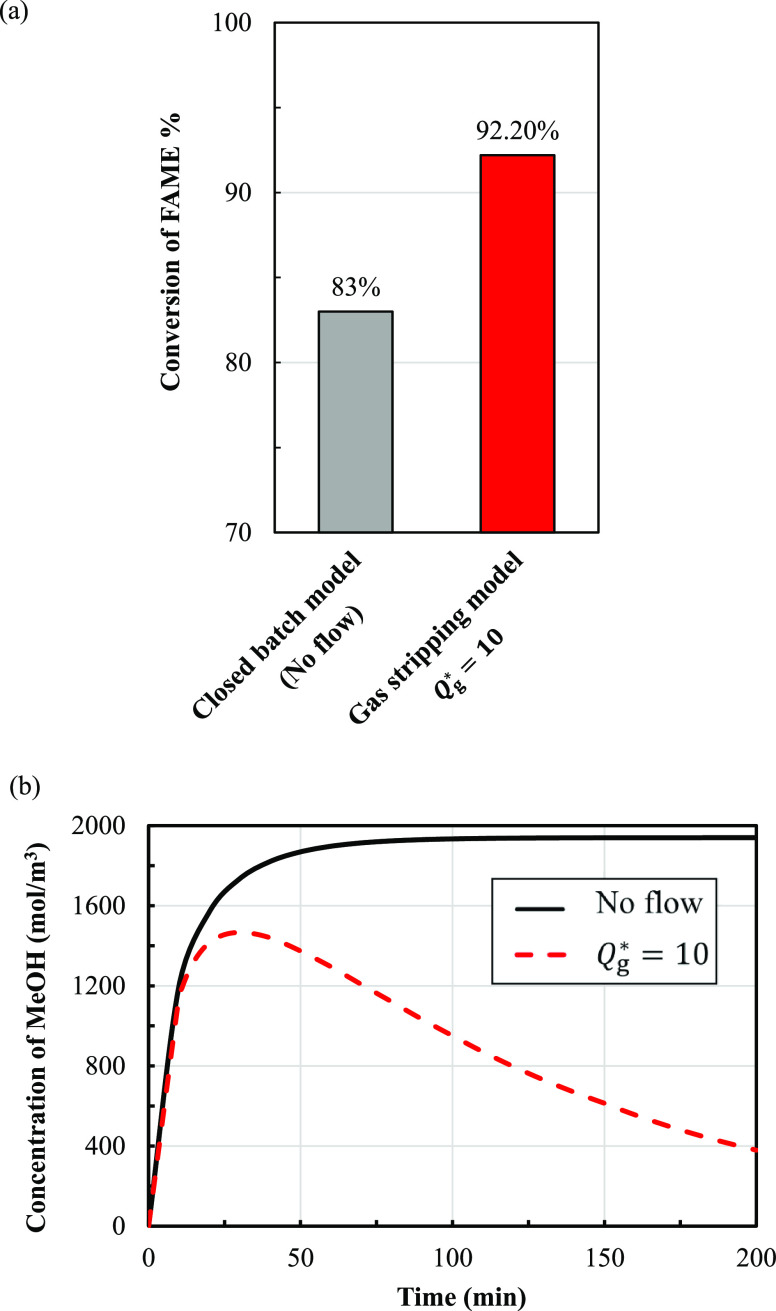
Comparison of the results for process intensification by gas stripping with *Q*_g_^*^ of 10 and for the base case with no gas flow: (a) FAME conversions and (b) concentration of methanol.

Figure 14a shows the comparison of the FAME conversions for the process intensification by gas stripping with *Q*_g_^*^ = 10 and that for the base case with no gas flow. After implementing the gas stripping, the FAME conversion is improved from 83 to 92.20%. Meanwhile, Figure 14b shows that the concentration of methanol in the liquid phase progressively decreases with time as gas stripping is implemented, thereby reducing the extent of the reverse reaction. However, during an initial period of up to 20 min, the gas stripping does not show any effect in methanol removal because not much methanol is produced in the liquid phase. Thus, the methanol concentrations in the liquid phase of the gas stripping case are, at least at the beginning, not much different from that of the closed batch case.

The analysis of process intensification also suggests the use of reactors, which has a high solute stripping efficiency such as bubble columns, gas-inducing agitated reactors, impinging jet reactors, and possibly the use of reactive distillation for a continuous operation.^21−25^

### 4.4 Results of Process Intensification by the Vacuum Stripping Model and Comparison with the Experiment

The calculation for process intensification by the vacuum stripping model is carried out using the pressure *versus* time plot, as shown in Figure 3. To obtain the target change of pressure over time, the exit volumetric flow rate of gas removal should be adjusted.

**Figure 15:**
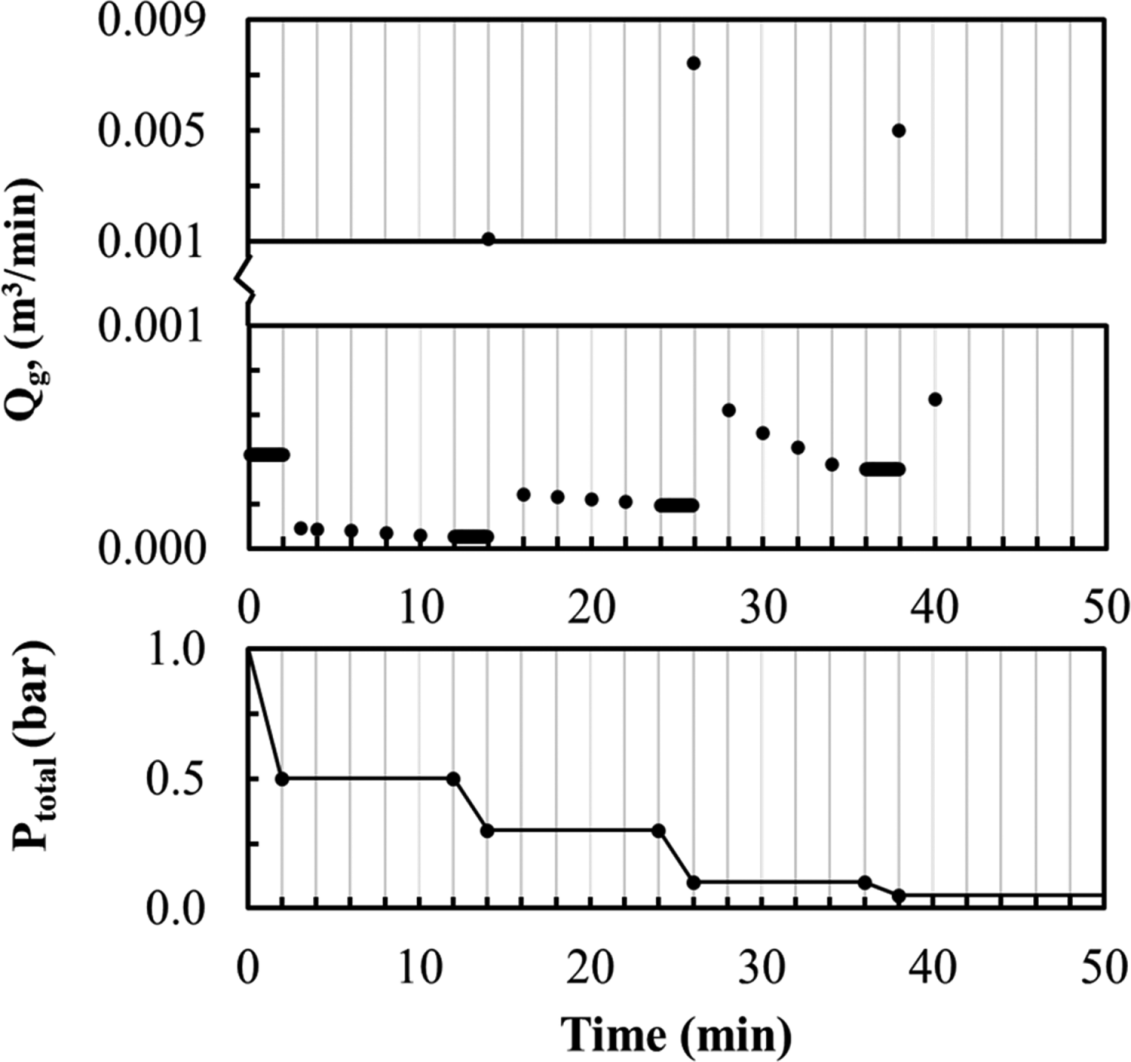
Obtained exit volumetric flow rate of gas removal (*Q*) over time corresponding to the required pressure drop plot (Figure 3).

The required pressure in the vacuum stripping, over short time intervals, must drop from one level to the next level. This pressure information is the target to be matched from solving eqs 39 and 40, in which the exit volumetric flow rate of gas removal (*Q*_g_) is the unknown variable. Over a fixed time interval, *Q*_g_ is obtained through optimization of eqs 39 and 40 to match the required pressure, which is the result of integration of eq 39. In addition to eqs 39, 40 and 33–36 are required. These four equations are used for solving *C*_MeOH_, which is the variable in eq 29. Figure 15 shows the exit volumetric flow rate of gas removal (*Q*_g_) over time obtained from optimization, which matches the pressure over the time plot shown in Figure 3.

The operating conditions used in the calculation of this section are shown in Table 6.

**Table 6:** Operating Conditions Used in the Experiment and Parameters Used for Calculation of the Rector with Vacuum Stripping

operating conditions in the experiment	
initial FAME concentration (mol/m^3^)	2509
initial DEA concentration (mol/m^3^)	2650
initial nitrogen partial pressure (bar)	1
initial methanol partial pressure (bar)	0
initial volume of the gas (mL)	900
initial volume of the liquid mixture (mL)	100
*M* = total reactor volume/initial volume of liquid mixture	10
temperature (°C)	80
Parameters used for the model	
*K*_eq_ at 80 °C (obtained from Section 4.2)	16.92
*k*_f_ at 80 °C (m^3^/mol·min) (obtained from Section 4.2)	3.95 × 10^–5^
γ_MeOH_ at 80 °C (obtained from Section 4.2)	0.7

During the total pressure being reduced from atmospheric pressure to 500, 300, 100, and 50 mbar, the corresponding exit volumetric flow rates of gas removal (*Q*_g_) are described in Table 7. Over a time interval, in which the pressure is decreased, the exit gas flow rate is held constant. On the other hand, over a time interval, in which the pressure is held constant, the exit gas flow rate is gradually decreased. The details of the changes of pressure and the corresponding exit gas flow rates over time are shown in Table 7.

**Table 7:** Change of Pressure and the Exit Volumetric Flow Rates of Gas Removal Over Time

time (min)	pressure drop	exit gas flow
0–2	from 1000 to 500 mbar	constant
2–12	held at 500 mbar	*Q*_g_ gradually decreased
12–14	from 500 to 300 mbar	Constant
14–24	held at 300 mbar	*Q*_g_ gradually decreased
24–26	from 300 to 100 mbar	Constant
26–36	held at 100 mbar	*Q*_g_ gradually decreased
36–38	from 100 to 50 mbar	Constant
38–293	held at 50 mbar	*Q*_g_ gradually decreased

**Figure 16:**
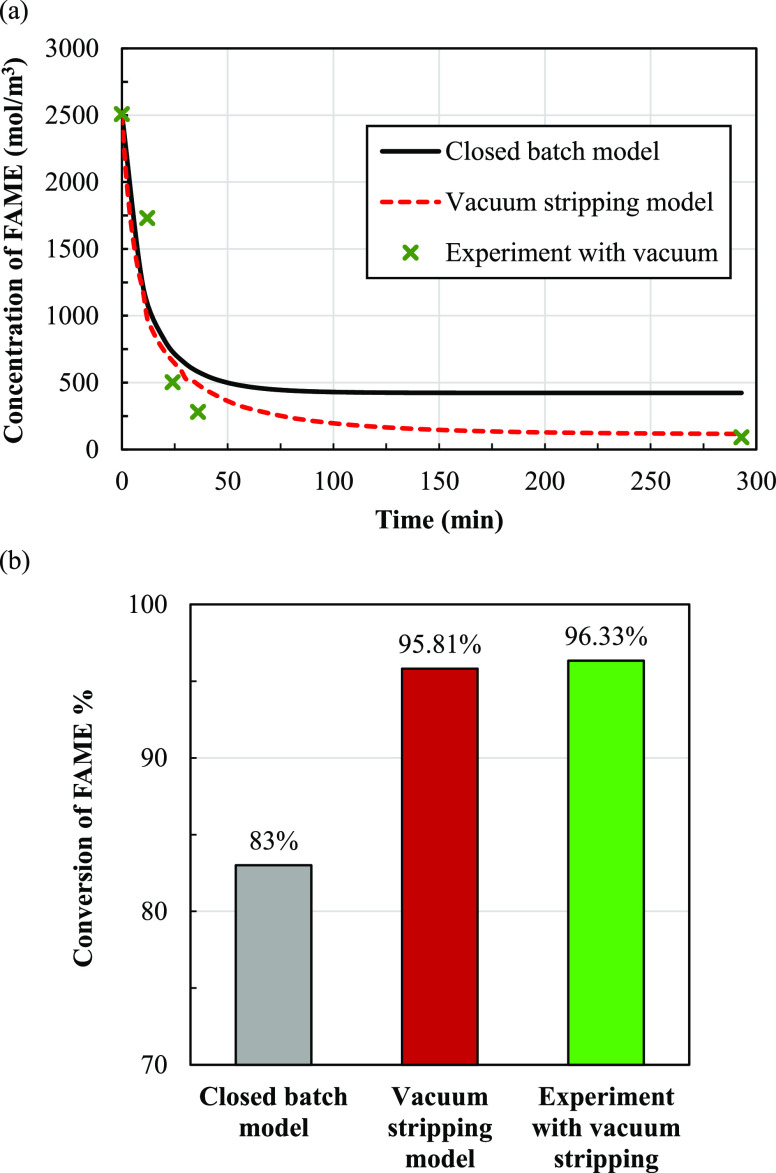
Effect of vacuum stripping on (a) change of the FAME concentration with time and (b) conversion of FAME; all are at a temperature of 80 °C.

The calculation results of the FAME concentration in process intensification of the vacuum stripping model are shown in Figure 16a. Comparing these results with those of a closed batch system, it was found that the conversion of FAME in the vacuum stripping model improves significantly due to methanol removal from the system. Indeed, the vacuum stripping model improves the FAME conversion from 83 to 95.81% (see Figure 16b). In addition, the change of the FAME concentration with time and the FAME conversion obtained from the calculations were compared with the experimental results using the same operating conditions, as shown in Figure 16. Calculated and experimental results are in good agreement.

## 5 Conclusions

The liquid-phase reaction of diethanolamine with a fatty acid methyl ester to produce fatty acid diethanolamide is reversible, with the final conversion being limited by the equilibrium. Manipulation to decrease the rate of the reversible reaction is required. Removal of the volatile product (*i.e*., methanol) is important to shift the reaction forward. During the reaction, methanol vaporizes from the reacting liquid phase to the nonreacting gas phase. Hence, the effect of mass transfer needs to be considered in the interpretation of kinetic data. The sensitivity of the overall model to the dimensionless mass transfer coefficient (α_lg_) is examined. When the dimensionless mass transfer parameter is greater than 4, the mass transfer model leads to an equilibrium model where the partial pressure of methanol in the bulk gas attains the same value as the partial pressure of the methanol at the interface. The results for α_lg_ = 4, which approaches the equilibrium model, are in good agreement with the experimental results. Using the equilibrium model, the rate and equilibrium constants were determined for the amidation of diethanolamine using experimental data in a batch reactor in the first part of this study. The value of the liquid activity coefficient at 70 °C was 0.8, while the values at 75 and 80 °C were found to be equal to one another and to 0.7. The forward and backward rate constant expressions were found to be 1.008 × 10^7^ exp (−9.28/*T*) (m^3^/mol·min) and 0.786 × 10^5^ exp (−8.56/*T*) (m^3^/mol·min), respectively. The expression of the equilibrium constant was determined to be ln *K*_eq_ = (0.716/*T*) + 4.855. It can be seen that the constant increased slightly with temperature, thereby indicating mild endothermicity.

The second part of the paper examines two process-intensification concepts—*viz*., inert gas and vacuum stripping of methanol from the reactor—and simulates the process based on mass-transfer-based models. Improvement in the final conversion was demonstrated for both approaches. Fatty acid methyl ester conversions for implementations of the inert gas stripping and vacuum stripping systems of methanol operated at 80 °C increased from a baseline value (a closed batch reactor result) of 83 to 92.2 and 95.81%, respectively. Finally, the predictions of the vacuum stripping model are in good agreement with the experimental results. Thus, the developed vacuum stripping model is useful for accurate analysis and design of a reactor with vacuum stripping.

The novelty of the work is obtaining the rate and reaction equilibrium constants, enthalpy of reaction, and the liquid activity coefficient for amidation, which has no prior reports and providing the viability of options for side product removal. The applied modeling approaches and the experimental facilities and methods are established.

## Nomenclature

*A*_lg_: gas–liquid interfacial area for mass transfer (m^2^)
*c*_*i*_: dimensionless molar concentration of species *i* in the liquid phase
*c*_MeOH,g_: dimensionless molar concentration of methanol in the gas phase
*C*_*i*_: molar concentration of species *i* in the liquid phase(mol/m^3^)
*C*_DEA_0__: Molar concentration of diethanolamine (mol/m^3^)
*C*_FAME_0__: molar concentration of the fatty acid methyl ester (mol/m^3^)
*C*_ref_: reference molar concentration in the liquid phase (mol/m^3^)
*C*_tot_: total molar concentration in the liquid phase (mol/m^3^)
*E*_b_: backward activation energy (kJ/mol)
*E*_f_: forward activation (kJ/mol)
*G*_f,*i*_: Gibbs free enthalpy of formation of species *i* (kJ/mol)
Δ*G*_rxn_: Gibbs free enthalpy change of reaction (kJ/mol)
*H*_f,*i*_: enthalpy of formation of component *i* (kJ/mol)
Δ*H*_rxn_: enthalpy of reaction (kJ/mol)
*H*_MeOH_: partition constant (Pa·m^3^/mol)
*H*_MeOH_^′^: dimensionless partition constant
*k*_b_: backward reaction rate constant (m^3^/mol·min)
*k*_f_: forward reaction rate constant (m^3^/mol·min)
*k*_g_: mass transfer coefficient from the liquid phase to the gas phase (m/min)
*K*_eq_: equilibrium constant (*k*_f_/*k*_b_)
*M*: total reactor volume per initial volume of the liquid mixture
MW_MeOH_: molecular weight of methanol (kg/mol)
*N*_MeOH_: moles of methanol in the liquid (mol)
*N*_tot_: total moles of methanol in the liquid (mol)
p_MeOH_: partial pressure of methanol in the gas phase (bar)
*p*_MeOH_^*^: partial pressure of methanol at the interphase (bar)
*p*_N_2__: partial pressure of nitrogen in the gas phase (bar)
*P*_tot_: total pressure (bar)
*P*_MeOH_^vap^: vapor pressure of pure methanol (bar)
*Q*_g_: volumetric flow rate of the gas (m^3^/min)
*Q*_g_^*^: dimensionless volumetric flow rate of the gas
*R*: gas constant (L·ba/mol·K)
*R*_FAME_: generation rate of the fatty acid methyl ester (mol/m^3^•min)
*s*_*i*_: stoichiometric number of species *i*
*t*: reaction time (minute)
*t*^***^: dimensionless time scale
*T*: temperature (K or °C)
*V*_g_: volume of the gas phase (m^3^)
*V*_l_: volume of the liquid phase (m^3^)
*V*_l_in__: initial volume of the liquid phase (m^3^)
*V*_R_: total volume of the reactor (m^3^)
*x*_MeOH_: mole fraction of methanol

## Greek letters

α_lg_: dimensionless mass transfer parameter
ε: liquid volume per initial liquid volume
γ_MeOH_: liquid activity coefficient of methanol
